# Combined splenectomy and robotic subtotal gastrectomy with short gastric vessel salvation for gastric cancer with SANT of the spleen: A case report

**DOI:** 10.1016/j.ijscr.2025.111558

**Published:** 2025-06-23

**Authors:** Hyojin Lee, Yoo Min Kim

**Affiliations:** a Yonsei University College of Medicine, 50-1, Yonsei-Ro, Seodaemun-gu, Seoul 03722, Republic of Korea; b Department of Surgery, Yonsei University College of Medicine, 50-1, Yonsei-Ro, Seodaemun-gu, Seoul 03722, Republic of Korea

**Keywords:** Gastric cancer, Robotic gastrectomy, Splenectomy, Real-time, Image-guided surgery, Case report

## Abstract

**Introduction and importance:**

Surgical methods to treat gastric cancer are quite straightforward. However, in rare cases of gastric cancer accompanied by a splenic mass that requires splenectomy, treatment options become much more complicated. Splenectomy can effectively treat splenic masses, but without adequate salvation of vessels, could increase the risk of ischemic necrosis if simultaneously performed with distal subtotal gastrectomy.

**Case presentation:**

The patient is a 40-year-old male diagnosed with cancer at the stomach angle. Abdominal CT also confirmed a huge mass in the spleen, suspected to be sclerosing angiomatoid nodular transformation (SANT). 3-dimensional CT angiography and RUS™ software were used to visualize the patient's intraabdominal anatomy before and throughout surgery. The robotic approach was used to incorporate said technologies while intricately preserving the short gastric vessels. Indocyanine green was injected intravenously to confirm adequate perfusion to the remnant stomach.

**Clinical discussion:**

Subtotal gastrectomy can be performed concurrently with splenectomy if the splenic and vascular anatomies allow for a safe splenectomy and preservation of the short gastric vessels. The feasibility of the operation should be confirmed by meticulous exploration of the patient's specific anatomy before and during surgery.

**Conclusion:**

This report demonstrates a successful method to perform subtotal gastrectomy with splenectomy. Despite its complexity and time-consuming nature, this procedure can greatly benefit patients by allowing safe resections and maximal preservation of digestive functions, nutrition, and quality of life. As a result, we recommend that it be more readily considered when treating complex cases like this.

## Highlights

• Combined splenectomy and subtotal gastrectomy increases risk of ischemic necrosis.
• RUS™ can provide intraoperative real-time guide of patient-specific vascular anatomy.
• RUS™ can help assess feasibility of combined splenectomy and subtotal gastrectomy.
• Indocyanine green injection should be used to confirm adequate perfusion.

## 1 Introduction

South Korea has one of the highest incidence rates for gastric cancer in the world [1]. Naturally, it is also one of the most prevalent types of cancer in the nation, accounting for about 10.8 % of all cancer incidences in Korea [2] and has remained a major public health burden for decades. Once detected, it is mostly treated with surgery as the first course of treatment [3]; lesions of the body or antrum are treated by subtotal gastrectomy, which involves resection of the gastric and gastroepiploic vessels while conserving the short gastric vessels. Sclerosing Angiomatoid Nodular Transformation (SANT) is a rare benign vascular proliferation of the spleen. Because it has no pathognomonic radiological features, histological confirmation is necessary to safely exclude malignancy; most patients are offered splenectomy instead of core biopsy to avoid the risk of hemorrhage, splenic rupture, and intraperitoneal seeding of malignancy [4]. We present a patient presenting with gastric cancer on the lower body of the stomach accompanied by a huge splenic mass, suspected to be SANT. All work has been reported in line with the SCARE criteria [5].

## 2 Case description

**Fig. 1:**
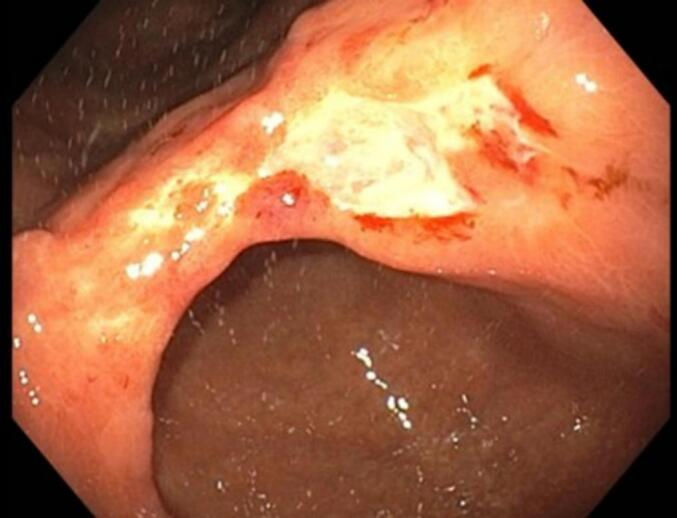
Esophagogastroduodenoscopy (EGD) finding of gastric cancer at stomach angle.

**Fig. 2:**
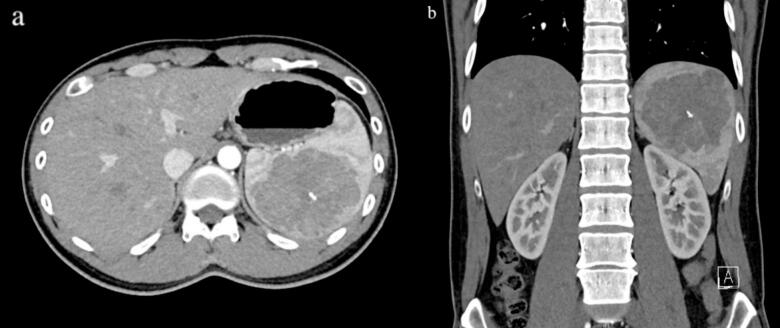
Abdominal CT: large splenic mass with intralesional calcification, r/o SANT (a) axial, (b) coronal view.

The patient is a 40-year-old male who was referred due to diagnosis of gastric cancer on his first ever National Cancer Screening esophagogastroduodenoscopy (EGD). The patient had no related symptoms or significant medical history. Endoscopy showed deep ulcerations and minor bleeding due to gastric cancer sized 3.0 × 2.0 cm (Fig. 1). Subsequent endoscopic biopsy showed poorly cohesive adenocarcinoma with signet ring cells at the stomach angle, and the pathological report diagnosed type IIa + IIc, making endoscopic resection impractical. *Helicobacter pylori* was present. CT angiography stomach pre-op showed T3/4a, due to few borderline-to-enlarged lymph nodes along the stomach lower curvature. Abdominal CT and liver MRI both confirmed an 8.6 cm well-defined lobulated splenic mass with intralesional calcification, likely being SANT, hamartoma, or other benign neoplastic lesions (Fig. 2a, b). However, the possibility of malignancy could not be completely ruled out and histologic confirmation was recommended.

**Fig. 3:**
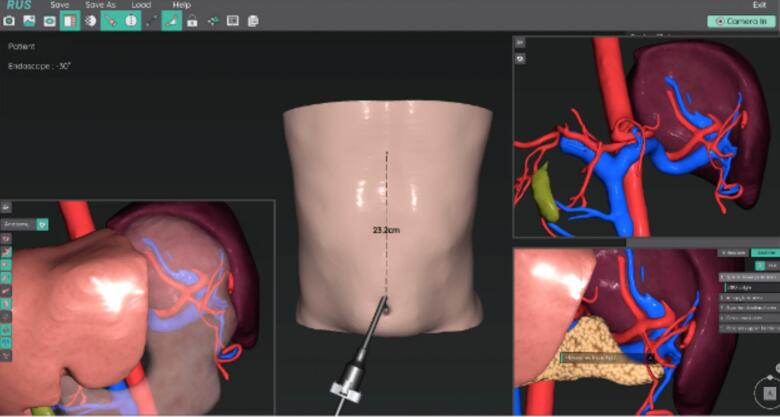
Preoperative port simulation using the RUS™ system: visualization of the patient's intraabdominal anatomy.

Because the gastric cancer was located at the angle, subtotal gastrectomy was the optimal treatment for the cancer itself. However, splenectomy was also necessary for the splenic mass. Performing both procedures simultaneously may increase the risk of post-operative complications such as ischemic necrosis of the remnant stomach [6], as standard splenectomy includes division of short gastric vessels [7]. Hence, we set the following treatment plans: if the spleen is safely resectable, perform total gastrectomy and splenectomy; if the spleen isn't safely resectable due to short gastric vessel anatomy, perform subtotal gastrectomy only and plan splenectomy for later (once collateral vessels to the remnant stomach develop). In addition, we decided to closely explore the vessel anatomy and assess whether it would be possible to attempt subtotal gastrectomy with splenectomy. This was because the patient was relatively young, and we wanted to conserve his postoperative digestive functions, nutrition, and quality of life as much as possible. To maximize this possibility, we decided to perform the robotic approach with intraoperative vessel navigation using preoperative 3-dimensional CT angiography and RUS™ software to visualize the patient's vascular anatomy and safely preserve the short gastric vessels [8]. The CT images recreated 3D structures of the patient's stomach and its surrounding vessels, allowing for preoperative simulation (Fig. 3). These images were accessed throughout surgery via RUS™ software connected to the robot system. Splenic biopsy prior to surgery was not done because of its high risk of bleeding [4], which would make total gastrectomy inevitable. On the day before surgery, indocyanine green (ICG) was endoscopically injected on the peritumoral submucosa to secure adequate resection margin [9] and identify the tumor's lymphatic flow for complete lymphadenectomy [10].

**Fig. 4:**
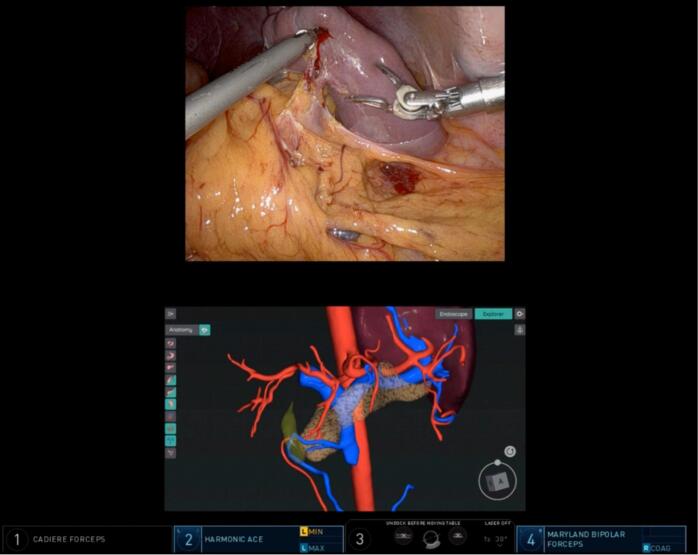
Intraoperative real-time guide of 3D vascular anatomy assisted by RUS™ system.

**Fig. 5:**
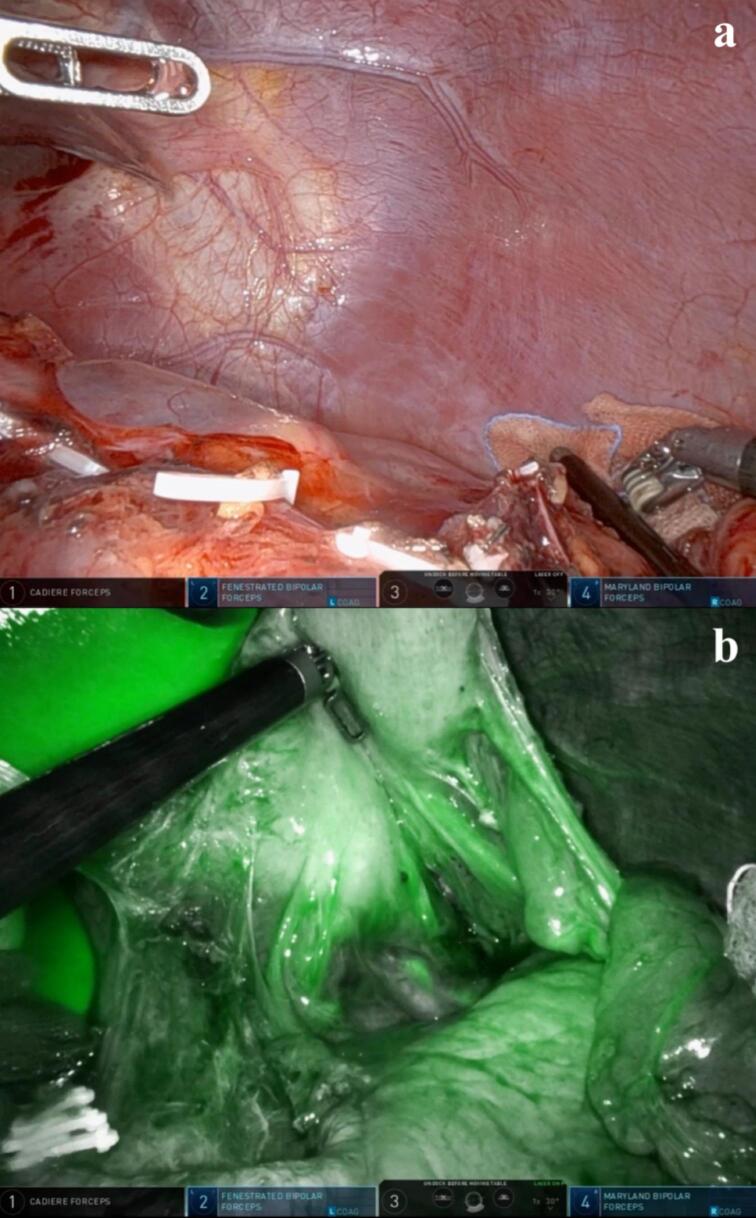
(a) View of left intraperitoneal space after splenectomy (b) Firefly fluorescence imaging of intravenous ICG shows adequate perfusion to remnant stomach via short gastric vessels.

The patient underwent standard subtotal gastrectomy, including partial omentectomy and D2 lymph node resection (Video 1). Right and left gastroepiploic vessels and right and left gastric vessels were dissected. Splenic end vessels were cautiously dissected and ligated very close to the spleen so that the short gastric vessels that supply blood to the stomach were not damaged. 3D CT angiography reconstruction assisted this process by providing real-time guide of the patient's vessel structure (Fig. 4). As we moved up along the spleen, the splenic end vessels were located more closely to each other and the stomach, hence were resected with more caution and time. Once splenectomy was completed (Fig. 5a), we intravenously injected ICG and examined the dye flow on Firefly fluorescence imaging mode to ensure adequate perfusion to the remnant stomach by short gastric vessels (Fig. 5b) [11]. Postoperative pathological diagnosis confirmed early gastric cancer at the middle third of the stomach, lesser curvature, type IIb, 3.0 × 2.0 cm in size. Resection margins and all 42 regional lymph nodes were free of carcinoma. The splenic mass was confirmed as SANT, 8.0 × 7.0 cm in size. The patient's final TNM stage for gastric carcinoma was T1aN0M0. The patient recovered without significant complications and was discharged on postoperative day 7 and vaccinated within 2 months. In his 1-year follow-up, no postoperative complications or recurrence were reported and nutritional status was adequate.

## 3 Discussion

Subtotal gastrectomy is a very common and regularly performed surgery for gastric cancer, but not so much in combination with splenectomy, as excessive resection of the short gastric vessels can induce gastric remnant necrosis. In our case, intraoperative findings allowed for combined subtotal gastrectomy with splenectomy due to minimal adhesions at the peri-splenic area, unchallenging spleen mobilization, and precise identification of the splenic end vessel anatomy.

In a case of combined subtotal gastrectomy and splenectomy for gastric cancer and possible spleen metastasis by Lee et al. [12], the spleen and short gastric arteries were completely removed, and the left gastric artery was preserved. In our patient, however, it was impossible to preserve the left gastric vessels as the gastric cancer was located at the angle. In another case of combined subtotal gastrectomy and splenectomy for gastric cancer and immune thrombocytopenic purpura (ITP) by Kaneko et al. [13], gastric remnant necrosis was prevented by preserving the ascending branch of the left gastric artery, the short gastric arteries, the posterior gastric artery, and the left gastroepiploic artery. SANT of the spleen is typically treated with total or partial splenectomy, depending on the anatomy of the spleen and size of the mass, and recurrence is rare [14]. In our patient, the sheer size of the tumor required a total splenectomy.

Thorough preoperative preparation and intraoperative techniques can help provide patient-tailored treatment in complex cases like this. Preoperatively, 3D CT angiography reconstruction provided a critical view of the vessel structure. Intraoperatively, real-time guide of the patient's vascular anatomy using RUS™ software allowed for easier vessel navigation. The RUS™ software has proven its accuracy and stability in patient-specific surgical navigation through a study including 30 gastric cancer patients undergoing gastrectomy by two surgeons [8], accounting for variations in patient anatomy, tumor characteristics, and surgical skills. The robotic approach maximized intricacy and stabilized tremor when handling the miniscule arteries during splenectomy. Such stability would not have been replicable through an open or laparoscopic approach. In addition, it enabled the display of multiple digital sources on the console screen via The TilePro™ function of the daVinci Xi system, allowing for flexible referral to the reconstructed anatomy [8]. Intravenous ICG injection allowed real-time visualization of blood flow to the remnant stomach via short gastric arteries. A combination of the above techniques resulted in the best functional outcome possible, while safely resecting both gastric cancer and splenic tumor. One limitation to our study is that because it is confined to a single case, it is difficult to conclude that RUS™ will always be useful to this degree in other cases. However, it is clear that this approach can benefit such complex cases by aiding in patient-specific treatment and making minimally invasive surgical approach safe and feasible.

## 4 Conclusion

This report demonstrates a successful method to perform subtotal gastrectomy with splenectomy by salvaging the short gastric vessels. Although it can be more complex and time-consuming compared to the traditional open or laparoscopic approach, the combination of 3D CT angiography and RUS™ software with robotic approach can greatly benefit patients by allowing safe resection and preserving maximal digestive function, nutrition, and quality of life. As a result, we recommend that it be more readily considered when treating complex cases like this.

The following is the supplementary data related to this article.

Video 1 Splenectomy using intraoperative real-time guide of 3D vascular anatomy assisted by RUS™ system and Firefly fluorescence imaging of intravenous ICG enabling real-time visualization of perfusion to remnant stomach via short gastric vessels. Video 1

## Author contribution

Hyojin Lee: responsible for conceptualization, drafting the manuscript and overseeing revisions.

Yoomin Kim: responsible for methodology and conceptualization, reviewing and providing supervision.

## Consent

Written informed consent was obtained from the patient for publication of this case report and accompanying images. A copy of the written consent is available for review by the Editor-in-Chief of this journal on request.

## Ethical approval

Ethics approval is pending from the institution's Institutional Review Board.

## Guarantor

Yoomin Kim.

## Research registration number

This is not a ‘First in Man’ study.

## Funding

This research did not receive any specific grant from funding agencies in the public, commercial, or not-for-profit sectors.

## Conflict of interest statement

There are no conflicts of interest to declare.
